# Can Tropical Africa Be Colonised?

**Published:** 1890-07-05

**Authors:** William Moore

**Affiliations:** K.C.I.E., Q.H.P., late Surgeon-General with the Government of Bombay


### Can Tropical Africa be Colonised?

By Sir William Moore, K.C.I.E., Q.H.P., late Surgeon-General with the Government of Bombay.

It will probably be interesting at the present time to consider the question whether or not equatorial Africa can be colonised by Europeans. Pictures have been word-painted of smiling European villages arising on African equatorial plains, and becoming the centres of civilisation and Christianity. The teeming populations of Europe were to find a new country, where colonists could obtain that livelihood from a fertile earth, on terms more easy than those offered them by the land of their birth.

Now, it is perfectly true that we do not possess any accurate or lengthened meteorological data regarding the climate of equatorial Africa. Still, we know pretty well what the coast climates are. The West Coast, extending from about N.L. 25 to S.L. 10, is perhaps the most unhealthy climate in the world. The East Coast, extending from the Red Sea to the centre of Madagascar, although not so unhealthy as the opposite coast, is still a very unhealthy locality. As a matter of fact, these coasts do not much differ as regards seasonal changes, rainfall, and temperature from the coasts of India. And we have sufficient experience of the Indian coast climates, demonstrating that Europeans cannot flourish there. But it will be said that it is the interior and higher lands which are fitted for the occupation of Europeans. But the interior and higher lands of tropical countries possess climates which only differ from the lowlands and coasts because the heat is tempered by elevation. The reduction of temperature attending elevation depends on numerous causes which need not be specified, and it is not the same on all elevations. One degree for every 250 feet is a good working average. Notwithstanding the reduction of heat—which is undoubtedly a great gain—there are the same seasonal changes; the hot, wet, and cold periods. There is the same vertical sun shining above. And in the interior of equatorial Africa there are two wet seasons, which culminate in eight months' rain. Now the principal cause of disease in a tropical climate is heat. For there is a maximum of heat which is most favourable to the well-being of both animals and vegetables. But if the heat rises above the maximum, the reverse is the case. Even in a temperate climate a season of extraordinary heat causes loss of appetite, languor, and debility by its depressant effect on the nervous system. There is, therefore, as the starting point of disease in the tropics, an approach to a condition with which we are familiar in the extraordinarily hot seasons of temperate climes. But in the tropics other influences are at work. A tropical atmosphere is rarefied by heat so that a given bulk of air contains less oxygen. Air expanded by heat is less elastic, and therefore less fitted to dilate the lungs. Owing to the lassitude produced by heat less exercise is taken, and breathing is therefore less accelerated by motion. It has also been proved that respiration is slower in a tropical climate. All this results in a diminished bulk of air being respired, and hence necessarily a smaller proportion of oxygen. There is still another cause of diminished supply of oxygen. The vitality of the red particles of the blood is lessened, and they become studded with refractory granules of a fatty nature. Dr. Brunton has pointed out that want of oxidation leads to the formation of fat, which probably takes place first in the imperfectly aërated blood, and this interferes with the function of the red particles as carriers of oxygen. Although the diminution of oxygen from all causes may be slight, *it is continuous*. As a result, the carbon expired is diminished; Dr. De Chaumont calculated by 25 p.c. of exhaled carbonic acid. But this is not all. Owing to increase of sensible and insensible perspiration, there is a smaller proportion of urine, so that there may not be a sufficient quantity of fluid to hold in solution all the effete matters which should be excreted by the kidneys and passed in the urine. The blood, therefore, becomes contaminated by waste material or its products. Such contamination lowers the vigour of the body, and renders it more liable to any disease; for nervous exhaustion and anæmia are established as first steps. Next comes that great liability to chill characterizing Europeans in tropical climates. The heat, by causing excessive action of the skin, and consequently cutaneous debility, renders the skin more impressionable than in Europe to falls of temperature, which are very extended in the tropics. There has been a disposition of late to ignore chill as a cause of disease in favour of microbes. But it is also admitted that a temporary depression caused by chill may afford germs the opportunity of development, when they would have been destroyed by inherent vital power had not chill interfered to lessen that power. However this may be, chill is quite sufficient to excite disease without germs, and especially in the debilitated system of the European in a tropical climate. The prevalence of a scorbutic taint in tropical climates, so-called malaria, disturbed sleep from various causes, an atmosphere saturated with moisture, and preventing evaporation from the skin, are other factors leading to the deterioration of Europeans in the tropics. Doubtless there are Europeans in tropical climates who, during many years, escape any decided blood deterioration as described above, but they are exceptions to the general rule. They are persons possessed of the suitable temperament and constitution, of careful habits of life, and who mostly have taken a change from the tropics whenever they could do so.

Now, if it were possible that a tropical country could be occupied, as Australia or Canada have been colonized, there would be some instances of the kind to be found. In Hindustan hundreds, or, indeed, thousands of Europeans, soldiers, pensioners, &c., have settled in or about the great Indian military stations. But there is not a great grandchild of pure European blood descended from any of these people in existence. There are numbers of Eurasians; but without the admixture of native blood the race dies out. Even in the hills, where the climate is of a more European character, it is the same. And it is an admitted fact that children brought up on the Indian hill ranges—females especially—have not the physique of children brought up in England, and are very liable to blood degeneration in after life. It is the same with the so-called Portuguese. There are no Portuguese in India (where they were once dominant, at least on the coasts) uncontaminated by native blood—excepting, of course, recent arrivals. And the Portuguese being inhabitants of a southerly, sunny land, might be thought better fitted for tropical colonisation than the northern Anglo-Saxon. I used above the phrase, "uncontaminated by native blood." But I used it in a conventional sense. For I do not think that native blood does contaminate, and in an article headed "The Eurasian Future," published some time ago in the "Indian Annals," I advocated the cause of the increasing Eurasian population of India, which eventually must become a great power in the State. It has been the custom to describe Eurasians as characterised by the vices of both Europeans and natives. This is unjust. The Eurasians of India require only fostering care to become characterised by the virtues of both Europeans and natives, rather than the reverse. And they are certainly much better fitted for a tropical climate than are pure Europeans. My attention has long been directed to the possibility of European colonisation in the tropics, and I quote from my work, published in 1862, entitled "Health in the Tropics, or Sanitary Art Applied to Europeans in India." "Colonists cannot live without hard labour. The constitution, the sensibility of the skin, and the habits of the European forbid such labour under a tropical sun. . . . Much of the hill side is rock, or too steep to be susceptible of cultivation, even by terrace work, and many of the hill valleys are so deadly malarious that the pioneers of cultivation and civilization would be destroyed before their efforts succeeded in transforming the valleys by drainage and culture into healthy localities. . . . The robust appearance of certain planters has been brought forward . . . . but it must be recollected that these gentlemen are not daily labourers. . . . their duties being those of inspectors and not the rough manual labour of the colonist. . . . . The fact is, for the white man and his offspring, there is no such thing as acclimatisation in India. As a rule Europeans enjoy the best of health, and suffer less from the heat during their first years of residence. Exposure, instead of 'hardening' the system, has the contrary effect, and the longer Europeans remain in the tropics the more they feel the effect of the tropical sun. . . . The deteriorating process, though slow, is nevertheless certain, and if acute dysentery, epidemic cholera, ardent fever, or sunstroke do not some day suddenly destroy, insidious malarious disease, cachexia loci, or splenic leucocythæmia sooner or later results." All this is certainly not favourable to the colonisation of tropical Africa by any European nation. The idea is a chimera which should not be taken into account. The influx of Europeans into Africa will be followed by the usual result when Europeans penetrate tropical and semi-tropical climates. A class of hybrids will arise. And the authorities should make that class from the first a special care; taking warning by the consequences of neglect, as displayed by the Eurasians of India.

